# Sparse Pd–Te Covalent Bridges Drive Anomalous Bulk‐to‐Monolayer Electronic and Magnetic Evolution in FePd_2_Te_2_

**DOI:** 10.1002/advs.75957

**Published:** 2026-06-03

**Authors:** Huaiyuan Zhao, Jianwen Fang, Mohan Luo, Yinuo Ye, Yulin Wang, Jianhui Yang, Hongwen Zhang, Peng Cheng

**Affiliations:** ^1^ Quzhou University Quzhou P. R. China; ^2^ Key Laboratory of Advanced Fuel Cells and Electrolyzers Technology of Zhejiang Province Ningbo Institute of Materials Technology and Engineering Chinese Academy of Sciences Ningbo Zhejiang P. R. China; ^3^ Key Lab of Materials Physics Anhui Key Lab of Nanomaterials and Nanotechnology Institute of Solid State Physics HFIPS Chinese Academy of Sciences Hefei P. R. China; ^4^ Beijing Key Laboratory of Opto‐electronic Functional Materials & Micro‐Nano Devices School of Physics Renmin University of China Beijing P. R. China

**Keywords:** dimensionality‐driven evolution, FePd_2_Te_2_, non‐vdW layered magnets

## Abstract

Non‐van‐der‐Waals (non‐vdW) layered magnets provide bonding degrees of freedom beyond dispersion‐dominated stacks, yet how dilute interlayer covalent bridges influence bulk‐to‐monolayer evolution remains insufficiently understood. Using first‐principles calculations, we investigate FePd_2_Te_2_ (FPT), a layered ferromagnet with sparse interlayer Pd─Te bridges. These bridges exhibit a covalent component, while the cleavage energy remains in the vdW‐like range (∼0.51 J·m^−2^), establishing FPT as an exfoliable non‐vdW layered magnet. Upon thinning from bulk to monolayer, local Fe moments and intralayer exchange terms increase slightly, whereas the Curie temperature decreases from 173 to 27 K. Analysis of multiplicity‐weighted exchange contributions indicates that this suppression of T_C_ is governed primarily by the weakening and eventual disappearance of interlayer ferromagnetic exchange J_3_, with enhanced antiferromagnetic competition from J_2_ providing an additional destabilizing contribution. Meanwhile, orbital reorganization near the Fermi level sustains a relatively large easy‐plane magnetocrystalline anisotropy (∼1.86 meV per Fe in bulk) with a moderate enhancement (∼8.6%) in the monolayer, gives rise to a dimensionality‐dependent reversal of magnetoelastic response under biaxial strain, and leaves the near‐E_F_ spin polarization comparatively robust up to ±5% strain. These results identify FPT as a representative exfoliable non‐vdW magnet and clarify how sparse interlayer covalent bridges govern its thickness‐dependent magnetic evolution.

## Graphical abstract

Bulk FePd_2_Te_2_ contains sparse interlayer Pd–Te covalent bonds, giving it unexpectedly low exfoliation energy and enabling van der Waals‐like exfoliation. Cleaving these bonds during exfoliation makes the monolayer magnetically distinct from the bulk: magnetic anisotropy energy increases, and the strain‐response coefficient of the magnetic moment changes sign, revealing unusual dimensional control in a layered magnet.

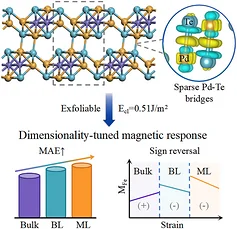

## 1 Introduction

Since the isolation of graphene, thinning layered solids from the bulk limit to the atomically thin regime has become a powerful route to emergent electronic, optical, and magnetic phenomena [1]. In magnetic materials, this dimensional reduction is especially significant because long‐range order, magnetic anisotropy, and spin‐dependent electronic structure are governed by a delicate interplay of exchange pathways, orbital hybridization, symmetry, and thermal fluctuations [2, 3]. Representative two‐dimensional magnetic platforms now include Cr_2_Ge_2_Te_6_ [4], CrI_3_ [5], Fe_5_GeTe_2_ [6], NiPS_3_ [7], Cr‐based MXenes [8, 9], and DyOCl [10]. Reducing thickness is therefore not merely a structural operation, but a means of reorganizing the microscopic interactions that determine functional response. For example, MoS_2_ evolves from an indirect‐gap to a direct‐gap semiconductor upon thinning [11], while CrI_3_ and related van der Waals magnets exhibit pronounced thickness‐ and field‐dependent magnetic behavior [5, 12]. Understanding how these interactions evolve from bulk to monolayer is thus important for both low‐dimensional magnetism and the design of ultrathin spintronic materials [1, 2].

Most current understanding of thickness‐dependent magnetism has been developed in van der Waals (vdW) materials [2, 3], where adjacent layers are coupled mainly by weak dispersion forces. In such systems, exfoliation typically weakens interlayer coupling while leaving the intralayer framework largely intact [2, 3]. A less explored situation arises in non‐vdW layered solids [13], where neighboring layers are connected by sparse yet directional chemical bonds. Particularly, non‐vdW ferromagnets that remain mechanically accessible in the few‐layer limit are still rare, further limiting a systematic understanding of bonding‐governed dimensional evolution in this class [13]. In these materials, dimensional reduction can remove specific bonding channels, modify local coordination, and redistribute near‐Fermi electronic states, potentially leading to thickness‐dependent behavior that differs from the conventional vdW picture [13]. How such dilute interlayer bonding influences the bulk‐to‐monolayer evolution of layered magnets, therefore, remains insufficiently understood.

FePd_2_Te_2_ (FPT) provides an appealing platform for addressing this issue. Recent experiments identified FPT as a layered ferromagnet with a quasi‐two‐dimensional crystal structure and mechanical exfoliability down to the few‐nanometer scale [14]. Beyond its anisotropic ferromagnetism, subsequent studies have also revealed unusual THz‐emission behavior in FePd_2_Te_2_‐based heterostructures [15], strain‐sensitive magnetic states [16], and spin‐polarized Dirac points with near‐flat bands [17], underscoring its broader interest as a layered magnetic platform. At the same time, crystallographic analysis revealed unusually short interlayer Pd─Te contacts (∼2.74 Å), substantially shorter than expected for a purely vdW gap, suggesting sparse interlayer bonding channels with a covalent component [14]. This combination raises a primary question: can FPT be understood as an exfoliable non‐vdW layered magnet, in which electronically active interlayer Pd─Te bridges coexist with a relatively low cleavage energy? A related question is how weakening or removing these bridges upon thinning is reflected in the evolution of its electronic and magnetic properties.

Here, we address FePd_2_Te_2_ from this perspective using first‐principles calculations. We first show that FPT is an exfoliable non‐vdW layered ferromagnet with sparse interlayer Pd─Te bridges that carry a clear covalent component while maintaining a cleavage energy within the vdW‐like range. We then examine how weakening these bridges upon thinning is accompanied by changes in the spin‐polarized electronic structure, exchange interactions, magnetic anisotropy, and strain response. By centering the bonding–exfoliation relationship and treating the thickness‐dependent magnetic evolution in that context, this work provides a clearer microscopic picture of FePd_2_Te_2_ as a representative exfoliable non‐vdW magnet.

## 2 Results and Discussion

### 2.1 Interlayer Pd─Te Covalent Bridges and Exfoliation Energetics

**FIGURE 1:**
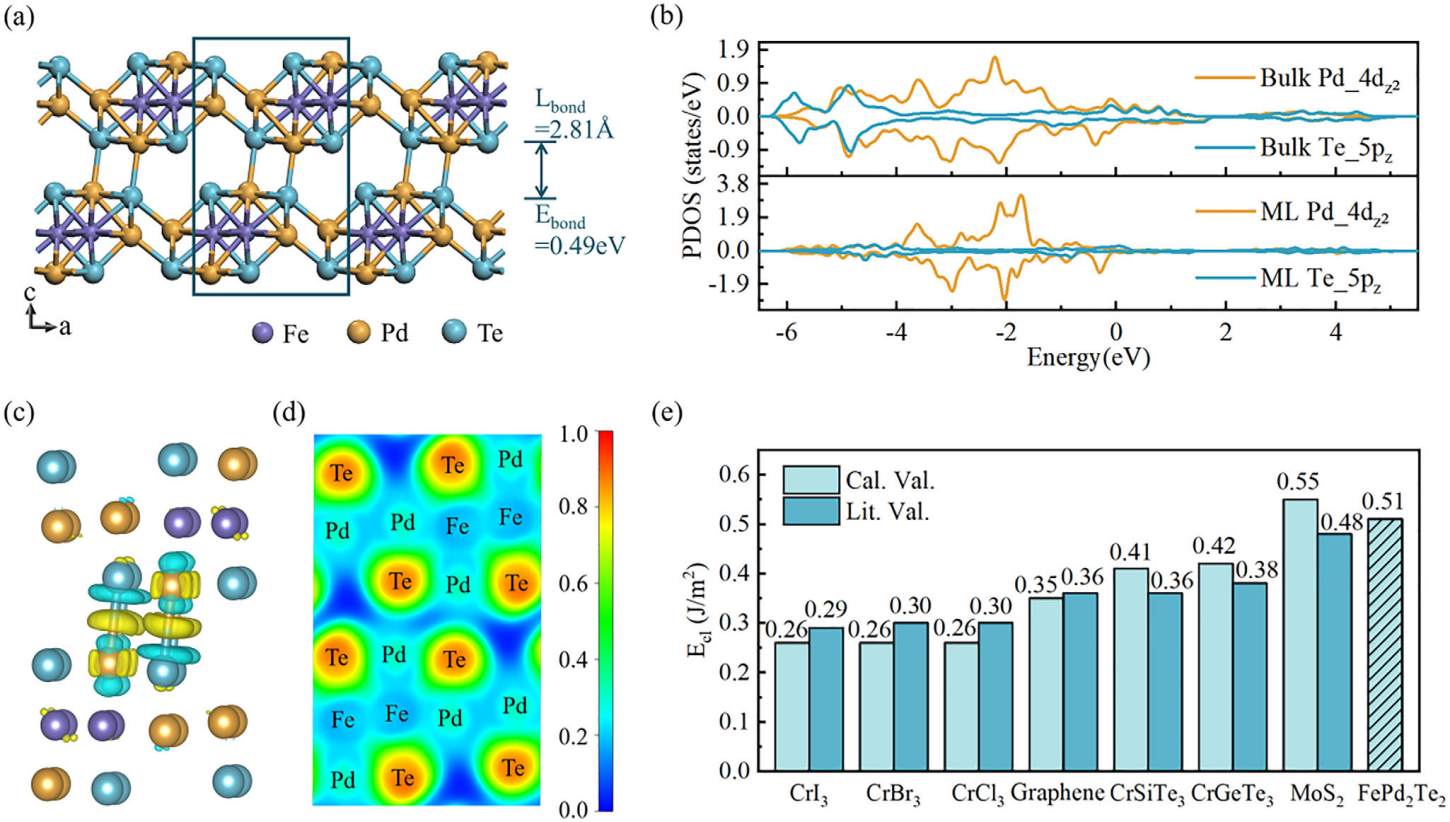
Structural, bonding, and exfoliation characteristics of FPT. (a) Side view of the bulk FPT structure. L_bond_ and E_bond_ denote the optimized Pd─Te bridge length and dissociation energy, respectively. (b) Projected density of states (PDOS) of the bridge‐forming Pd 4d_z_^2^ and Te 5p_z_ orbitals in bulk (solid lines) and monolayer (dashed lines) FPT. For direct comparison, the monolayer PDOS is normalized per atom. (c) Electron‐density‐difference map of bulk FPT, defined as Δρ =ρ_bulk_ ‐ρ_upper_‐ρ_lower_, where ρ_upper_ and ρ_lower_ are the electron densities of the isolated upper and lower structural fragments, respectively, calculated at the same atomic positions as in the bulk geometry. The isosurface value is 0.003 e·Å^−3^. Yellow and blue regions denote electron accumulation and depletion, respectively. (d) Electron localization function (ELF) map of bulk FPT in a plane containing the interlayer Pd─Te bridge. ELF values range from 0 (fully delocalized) to 1 (fully localized). (e) Comparison of cleavage energies E_cl_ among representative layered materials and FPT. Light bars denote values recalculated in this work under a common computational setup, while the dark bars are values extracted from the literature.

As evidenced by Table S1 and Equation S1, the proposed calibrated first‐principles protocol reproduces experimental lattice constants and Fe local magnetic moment with low relative errors (below 3%), as well as T_C_ values with a small deviation (only 5.5%), demonstrating the good reliability of our computational approach. In Figure 1a, the optimized FPT structure induces an interlayer Pd─Te bridge length of 2.81 Å, corresponding to only 2.6% longer than the experimental value of 2.74 Å. According to Equation S2, the corresponding bridge dissociation energy is estimated as 0.49 eV per Pd─Te bond. All these structural and energetic metrics indicate significantly stronger interlayer coupling than pure vdW interactions, carrying a substantial covalent component.

**FIGURE 2:**
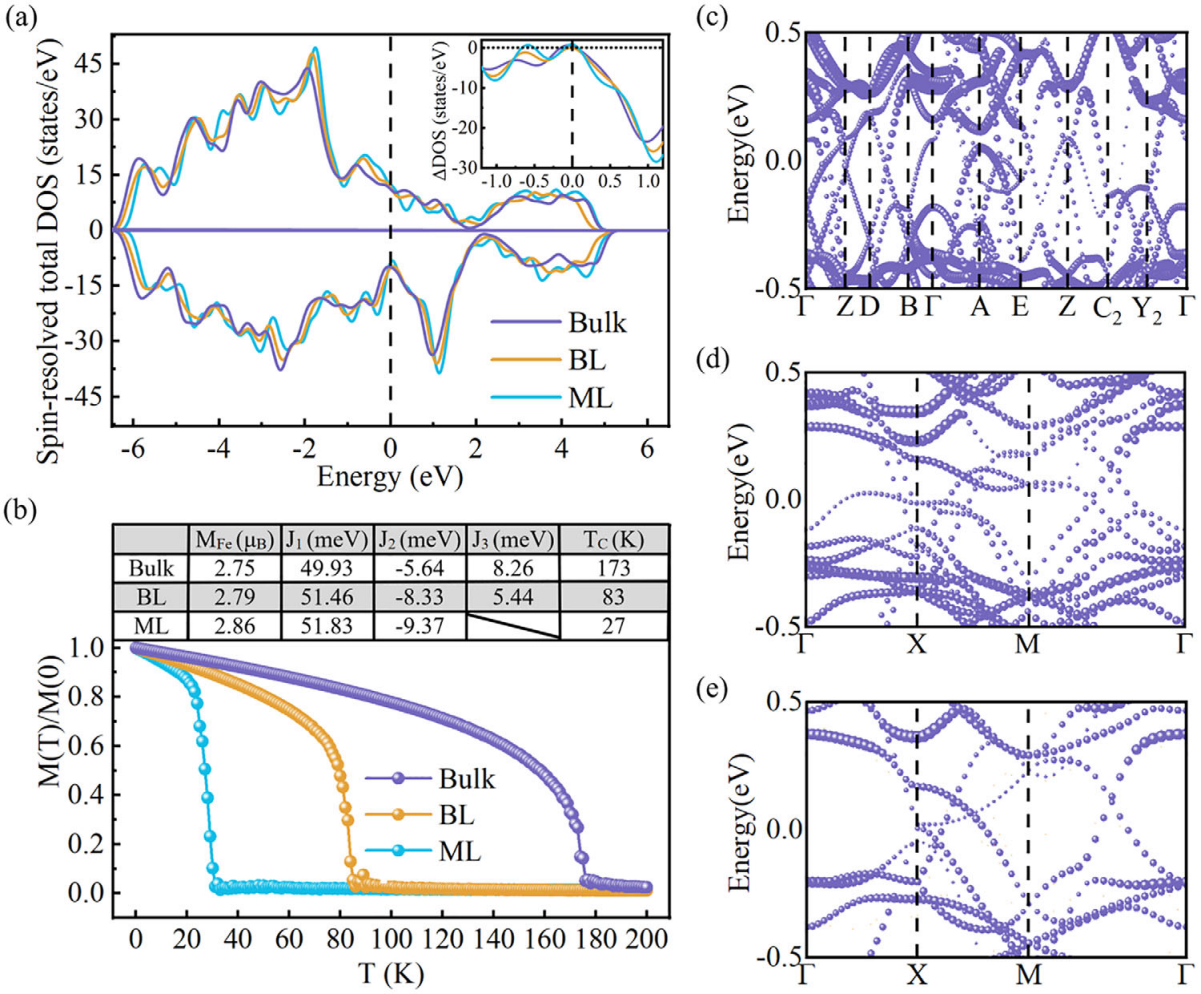
Dimensionality‐driven coevolution of electronic structure and magnetism in FPT. (a) Spin‐resolved total density of states (DOS) for bulk, BL, and ML FPT. The inset shows the energy‐dependent spin asymmetry Δ*D*(*E*)  = *D*_↑_  − *D*_↓_ in the vicinity of E_F_. (b) Normalized magnetization M(T)/M(0) as a function of temperature for bulk, BL, and ML FPT. The inset table summarizes key magnetic parameters: Fe magnetic moment (M_Fe_), exchange constants (J_1_, J_2_, J_3_), and the estimated T_C_. (c–e) Fe‐projected band structures for (c) bulk FPT along the three‐dimensional path Γ–Z–D–B–Γ–A–E–Z–C_2_–Y_2_–Γ, and for (d) BL and (e) ML FPT along the two‐dimensional path Γ–X–M–Γ.

As displayed in Figure 1b–d, the electronic signatures of the interlayer Pd─Te covalent bridges are evident in PDOS, electron redistribution, and ELF. In bulk FPT, the bridge‐forming Pd 4d_z_^2^ and Te 5p_z_ orbitals show strong energy alignment and PDOS overlap in both the bonding (−6 to −4 eV) and antibonding (−2 to 2 eV) regions, resulting in clear bonding–antibonding splitting. In the monolayer (ML), these hybridization features are strongly reduced, corroborating the disappearance of the interlayer bridge (Figure 1b). It should be noted that Figure 1b focuses specifically on the local orbitals associated with the interlayer Pd─Te bridge rather than on the full orbital manifold of the entire system. Because these bridge‐sensitive Pd/Te states account for only a relatively small fraction of the total DOS, their pronounced reorganization upon exfoliation does not necessarily produce equally dramatic changes in the full DOS shown in Figure 2a.

In Figure 1c, the electron‐density difference is defined as Δρ =ρ_bulk_ ‐ρ_upper_‐ρ_lower_, where ρ_upper_ and ρ_lower_ denote the electron densities of the isolated upper and lower structural fragments, respectively, calculated at the same atomic positions as in the bulk geometry. The resulting Δρ map reveals continuous electron accumulation across the Pd─Te bridge, accompanied by electron depletion on neighboring sites, indicating the occurrence of partial charge transfer and polarization along the bonding axis. The ELF cross‐section along the interlayer Pd─Te bond in Figure 1d displays a continuous mid‐range ridge (≈0.3–0.7) at the bond center, co‐located with the Δρ maximum (isosurface 0.003 e·Å^−3^), suggesting that bonding electrons are primarily distributed in the bridge region with a clear covalent‐sharing character. Meanwhile, high ELF values (>0.8) are recorded near Te nuclei, reflecting lone pairs, whereas the interlayer void retains a low ELF (<0.2), ruling out metallic‐like delocalization. All these observations support the presence of interlayer coupling mediated by a Pd─Te bridge with a significant covalent component.

However, despite the clear covalent component of the interlayer Pd─Te bridges, their areal density is low (∼0.13 Å^−2^, eight bonds per 63 Å^2^ cell; Figure 1a). As a result, the interlayer binding contribution per unit area remains moderate, yielding a calculated cleavage energy of only ∼0.51 J·m^−2^ for FPT (Figure 1e). This value is comparable to those reported for representative exfoliable layered materials, including chromium trihalides such as CrI_3_, CrBr_3_, and CrCl_3_ [18, 19, 20, 21, 22, 23], layered chromium tellurides such as CrSiTe_3,_ and CrGeTe_3_ [24, 25], as well as graphene [26] and MoS_2_ [27]. Accordingly, FPT can be considered an easily exfoliable layered magnet, although exfoliation from bulk to monolayer necessarily involves breaking sparse interlayer Pd─Te bonds. This unusual bonding topology, in turn, raises the central question of how dimensional reduction reshapes the magnetic response.

### 2.2 Dimensionality‐Driven Coevolution of Electronic Structure and Magnetism

The exfoliation of FPT from bulk to bilayer (BL) and monolayer for the spin‐resolved total DOS is compared in Figure 2a. A broad redistribution of the electronic states is observed, from deep valence states up to the vicinity of the Fermi level instead of a simple rigid‐band shift. The most pronounced changes take place between −1.5 and 0 eV, where states are expected to dominate transport. In the bulk, spin asymmetry looks relatively weak in the energy range of −1.5 to −0.7 eV, but becomes stronger between −0.7 and −0.3 eV. The exfoliation to the monolayer results in a partial reversal of the patterns, with strongly suppressed spin polarization in the −0.7 to −0.3 eV window, while an enhancement is recorded in the −1.5 to −0.7 eV range (inset of Figure 2a). Such a reshaping of the spin‐polarized spectrum can be attributed to the breaking of sparse interlayer Pd─Te covalent bridges and the consequent reorganization of near‐Fermi Pd─Te─Fe states, implying qualitative changes in spin‐dependent transport characteristics of FPT as a function of thickness. Therefore, the sparse Pd─Te bridges contribute directly to the near‐Fermi manifold, and their rupture upon exfoliation selectively reorganizes the spin‐polarized states that modulate electronic transport properties and magnetism, leading to a nontrivial thickness‐dependent evolution of the spin‐polarized electronic structure beyond a simple weak vdW picture.

The exchange parameters and T_C_ values were obtained by computing a set of competing magnetic configurations (ferromagnetic and several antiferromagnetic orderings) for bulk, bilayer, and monolayer FPT, and the resulting spin arrangements are gathered in Figures S1–S3. The optimized lattice parameters and the values of local Fe moment (M_Fe_) for these configurations are provided in Tables S2–S4. To obtain the nearest‐neighbor (J_1_), next‐nearest‐neighbor (J_2_), and interlayer (J_3_) exchange couplings, the obtained total energies were mapped onto a classical Heisenberg model, as detailed in Section S1. The thickness dependence of M_Fe_, the extracted exchange parameters, and the resulting T_C_ is summarized in Figure 2b. A clear monotonic decrease in T_C_ is observed from 173 K in the bulk to 83 K in the bilayer and 27 K in the monolayer, indicating progressively weakened long‐range ferromagnetic order upon dimensional reduction. At the same time, the exchange network is not rigid: J_1_ increases slightly from 49.93 to 51.83 meV, |J_2_| increases from 5.64 to 9.37 meV, whereas the interlayer exchange J_3_ decreases from 8.26 meV in the bulk to 5.44 meV in the bilayer and vanishes in the monolayer. To assess their relative roles more quantitatively, we compare the multiplicity‐weighted exchange contributions entering the Heisenberg mapping, namely 2J_1_, 2J_2_, and 4J_3_ (Table S5). From bulk to monolayer, the ferromagnetic stabilization associated with 4J_3_ decreases from 33.04 to 0 meV, whereas the competing antiferromagnetic contribution 2J_2_ changes from −11.28 to −18.74 meV. By contrast, the increase in 2J_1_ is much smaller, from 99.86 to 103.66 meV. These trends indicate that the suppression of T_C_ arises from the combined effects of the progressive weakening and eventual disappearance of the interlayer ferromagnetic exchange J_3_ and the enhanced antiferromagnetic competition associated with J_2_. Quantitatively, the reduction in the stabilizing 4J_3_ term is substantially larger than the change in the competing 2J_2_ term, indicating that the loss of J_3_ is the dominant factor, while the strengthening of |J_2_| provides an additional destabilizing contribution. Thus, exfoliation progressively breaks the three‐dimensional exchange network in FPT, reducing it first to a quasi‐2D network and ultimately to the monolayer limit. In this reduced‐dimensional regime, enhanced spin fluctuations can outweigh the modest strengthening of the in‐plane exchange channels, thereby contributing to the marked suppression of T_C_. This behavior is distinct from the conventional vdW picture, in which thickness reduction is often discussed mainly in terms of weakened interlayer coupling, while the accompanying reorganization of the intralayer exchange balance is usually less pronounced. In FPT, by contrast, the suppression of T_C_ is accompanied not only by the disappearance of J_3_ but also by measurable changes in J_2_, J_1_, and M_Fe_.

The comparison of the Fe‐projected band structures for bulk, bilayer, and monolayer FPT in Figure 2c–e confirms the electronic reconstruction upon dimensional reduction. Bulk FPT, plotted along the three‐dimensional high‐symmetry path Γ–Z–D–B–Γ–A–E–Z–C_2_–Y_2_–Γ, exhibits strongly entangled Fe‐d bands with pronounced dispersion near the Fermi level (E_F_), reflecting the presence of a three‐dimensional exchange network. The reduction in thickness to bilayer and monolayer results in the collapse of the band path to the two‐dimensional trajectory Γ–X–M–Γ, lifting the features associated with k_z_ dispersion and degeneracies. This results in significantly reorganized Fe‐d bands near E_F_, with some branches shifting downward in energy, whereas others move closer to or even cross the E_F_, indicative of a non‐rigid reshaping of the near‐Fermi manifold instead of a simple band shift. The element‐resolved band structures for both spin channels in Figures S5–S10 confirm near‐E_F_ bands dominated by Fe‐d states with non‐negligible Pd‐d and Te‐p hybridization, corroborating the above inferred Pd─Te─Fe covalent network.

Compared to the bulk, several Fe‐d‐dominated bands within roughly −0.3 to 0 eV display reduced dispersion and locally flatter segments in the bilayer and monolayer along Γ–X and X–M. Based on the effective mass scale m*∝[*d* ^2^ *E*/*dk* ^2^]^−1^, such a weaker curvature implies heavier carriers and enhanced quasi‐two‐dimensional localization for corresponding states. As the thickness is reduced, the Fe‐d spectral weight gradually accumulates below the E_F_ (≈ −0.3–0 eV), consistent with the spin‐resolved DOS in Figure 2a. By contrast, part of the deeper Fe‐d weight is shifted away from E_F_. Such a concentration and partial flattening of Fe‐d states near E_F_ corroborate the enhanced local moments and intralayer exchange constants, indicating rupture of sparse interlayer Pd─Te covalent bridges not only reshaping the near‐Fermi band topology but also reinforcing the low‐energy magnetic and transport responses of ultrathin FPT.

Therefore, the dimensionality reduction in FPT is closely associated with its electronic and magnetic degrees of freedom, showing a good correlation between the partial flattening and redistribution of near‐E_F_ Fe‐d bands with enhanced local moments and intralayer exchange couplings, while breaking the sparse interlayer Pd─Te covalent bridges cuts off three‐dimensional exchange pathways and destabilizes long‐range order. Such coexisting strengthened local magnetism and weakened global ordering stability further support the important role of sparse interlayer covalent bridges in shaping the thickness‐dependent magnetic response of non‐vdW layered magnets and suggest their potential relevance for ultrathin magnetic materials design.

### 2.3 Dimensionality‐Driven Evolution of Magnetic Anisotropy

**FIGURE 3:**
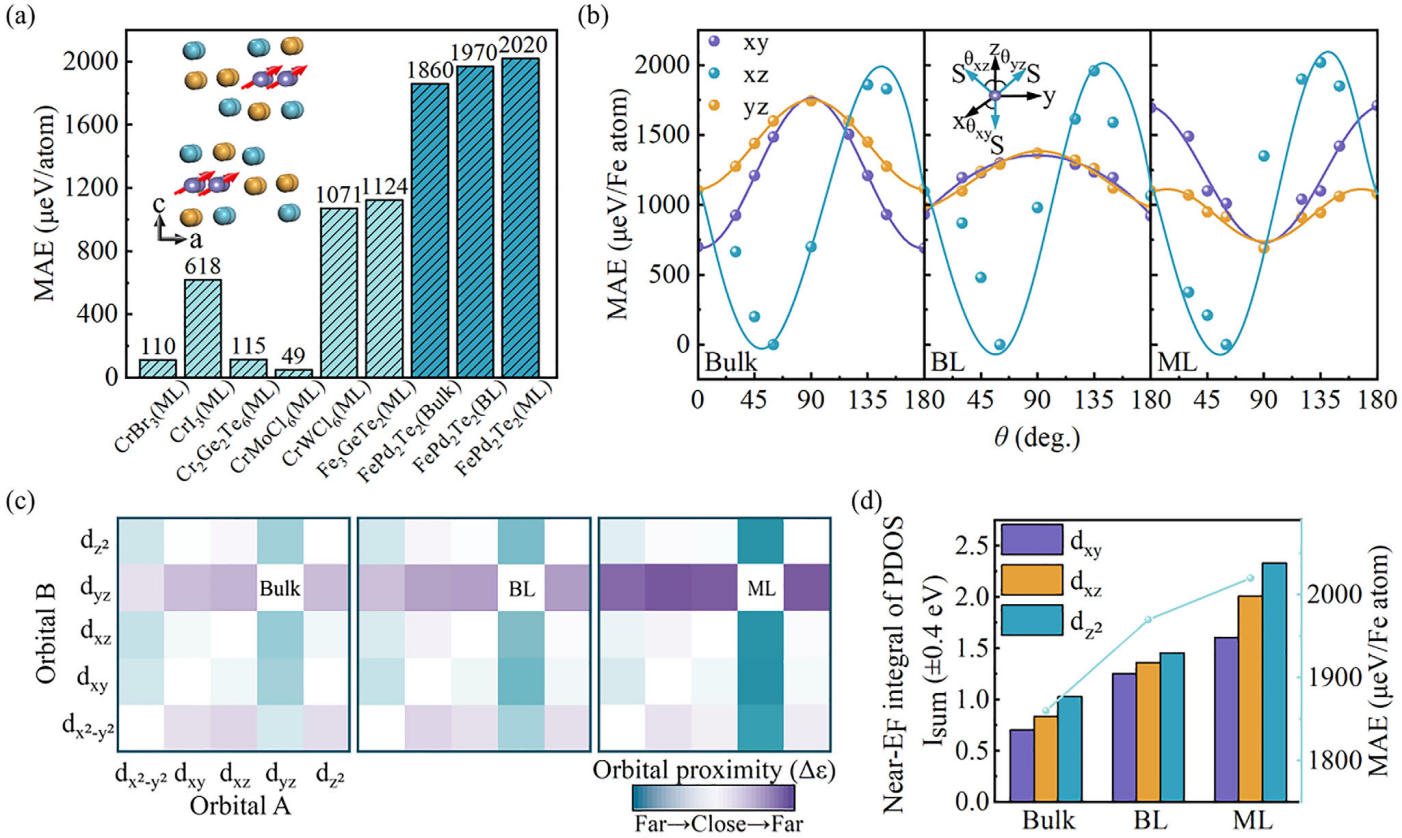
MAE and its orbital origin in FPT. (a) Calculated MAE values for FPT in bulk, BL, and ML form in comparison with several representative 2D magnets. (b) Angular dependence of the MAE in bulk, BL, and ML FPT for magnetization rotated within the xy, xz, and yz planes. (c) “Orbital proximity” maps Δε between occupied (orbital A) and unoccupied (orbital B) Fe‐d states, obtained from PDOS centroids within ±0.4 eV of E_F_ (Equations S11–S13). (d) I_sum_ for selected Fe‐d orbitals (d_xy_, d_xz_, and d_z_^2^) evaluated within ±0.4 eV (bars), together with the corresponding MAE per Fe atom (line, Equation S14).

In bulk (Figure 3a), FPT exhibits a relatively large magnetic anisotropy energy (MAE), with easy‐plane MAE reaching 1.86 meV per Fe atom, a value about 1–2 times larger than those of typical reported prototypical vdW magnets (such as CrBr_3_ and CrMoCl_6_) and exceeding high‐MAE systems (such as CrWCl_6_ and Fe_3_GeTe_2_). Such a large MAE imposes a high energetic cost on magnetization reorientation, which is favorable for maintaining magnetic anisotropy against thermal fluctuations.

Upon dimensional reduction, the easy‐plane MAE increases to ≈1.97 and ≈2.02 meV per Fe atom in the bilayer and monolayer, equivalent to about 6% and 9% enhancement relative to the bulk, respectively. This thickness‐enhanced MAE contrasts with the behavior commonly discussed for conventional vdW magnets, where exfoliation from bulk to monolayer usually results in nearly no changes in MAE (such as in CrI_3_ or Fe_3_GeTe_2_) or even weakening to drive easy‐plane transitions (such as in Cr_2_Ge_2_Te_6_) [23, 25, 28]. Such a robust anisotropy with only moderate thickness enhancement suggests the dominant contribution to the MAE originates from the intralayer Fe─Te─Pd coordination environment, whereas the sparse interlayer Pd─Te bridges act as stabilizers of the three‐dimensional magnetic order without strongly constraining the anisotropy. Thus, breaking the bridges upon exfoliation does not reduce the MAE but slightly enhances it, a less commonly discussed behavior among 2D magnets, suggesting the potential of non‐vdW layered systems such as FPT for ultrathin magnetic devices.

The angular dependence of the MAE upon rotating the magnetization within the xy, xz, and yz planes for bulk, bilayer, and monolayer FPT is highlighted in Figure 3b. For all thicknesses, the MAE varies smoothly with a 180° periodicity, indicating an anisotropy dominated by conventional second‐order spin–orbit coupling. The largest modulation is always in the xz plane, with an energy difference between the easy and hardest directions approaching 2 meV per Fe atom, defining a robust easy axis within this plane. By contrast, rotations within the xy and yz planes produce much smaller variations and become flatter with the decrease in thickness. Notably, the xy‐plane curve exhibits an ∼180° phase shift from bulk to monolayer, indicating that the in‐plane easy and hard axes interchange. This trend is consistent with the dimensionality‐driven redistribution of Fe─Te─Pd orbital weights discussed below.

The MAE microscopically originates from spin–orbit coupling (SOC). Based on Bruno's second‐order perturbation, the SOC can be used to link the spin orientation to the orbital angular momentum, and MAE can be expressed between two magnetization directions α and β:

(1) $$K_{\alpha \beta }\approx \xi^{2}\sum_{o\in \;occ\;}\sum_{u\in \;unocc}\frac{{\left|\langle o\left|L_{\alpha }\right|u\rangle \right|}^{2}-{\left|\langle o\left|L_{\beta }\right|u\rangle \right|}^{2}}{\varepsilon_{u}-\varepsilon_{o}}$$

where ξ refers to the SOC constant, *o* and *u* label occupied and unoccupied states, and ε*_u_*−ε*_o_* is their energy separation.

In this framework, the MAE is approximately proportional to the anisotropy of the orbital moment, Δ *m_L_* = *m*_*L*(α)_ ‐*m*_*L*(β)_. For FPT, the angular scans in Figure 3b show the global easy axis always lying within the xz plane for all thicknesses, identifying L_x_ and L_z_ as relevant competing components, with L_z_ providing the dominant contribution.

To gain a better understanding of the connection between this proposed orbital picture and the calculated MAE, further calculations were performed to quantify how dimensionality reorganizes Fe‐d levels and weights near E_F_. The obtained computational details are provided in Section S2. To this end, PDOS‐weighted energy centroids were used to estimate the level spacing Δε(A→B) between selected occupied and unoccupied Fe‐d orbitals [Equations (S11)–(S13)]. In the bulk and especially in the bilayer, the smallest Δε mainly involves the d_yz_ orbital (Figure 3c). In the monolayer, the closest pairs shift to combinations of d_z_^2^ with in‐plane d_xy_ and d_xz_ (Figure 3c), indicating a progressive strengthening of SOC‐active couplings between d_z_^2^ and d_xy_/d_xz_ in the 2D limit that effectively reduces the energy denominators in Equation (1).

Complementarily, the “numerator” of Equation (1) is characterized by a near‐E_F_ PDOS integral I_sum_(X) for each Fe‐d orbital X, counting the spectral weight within E_F_ ± 0.4 eV available for SOC‐mediated virtual excitations [Equation (S14)]. As illustrated in Figure 3d, I_sum_ for d_z_^2^ and d_xy_ increases markedly from bulk to bilayer and then to monolayer, tracking the monotonic rise of the MAE. Therefore, the reduced orbital gaps Δε between d_z_^2^ and d_xy_/d_xz_, as well as their enhanced near‐E_F_ spectral weight, both amplify the effective. ∣〈*o*|*L*_*x*,*z*_∣*u*〉|^2^/(ε_*u*_ − ε_*o*_) terms in Equation (1), microscopically explaining the strengthened easy‐plane MAE in ultrathin FPT.

### 2.4 Dimensionality‐Driven Magnetoelastic Coupling in FPT

**FIGURE 4:**
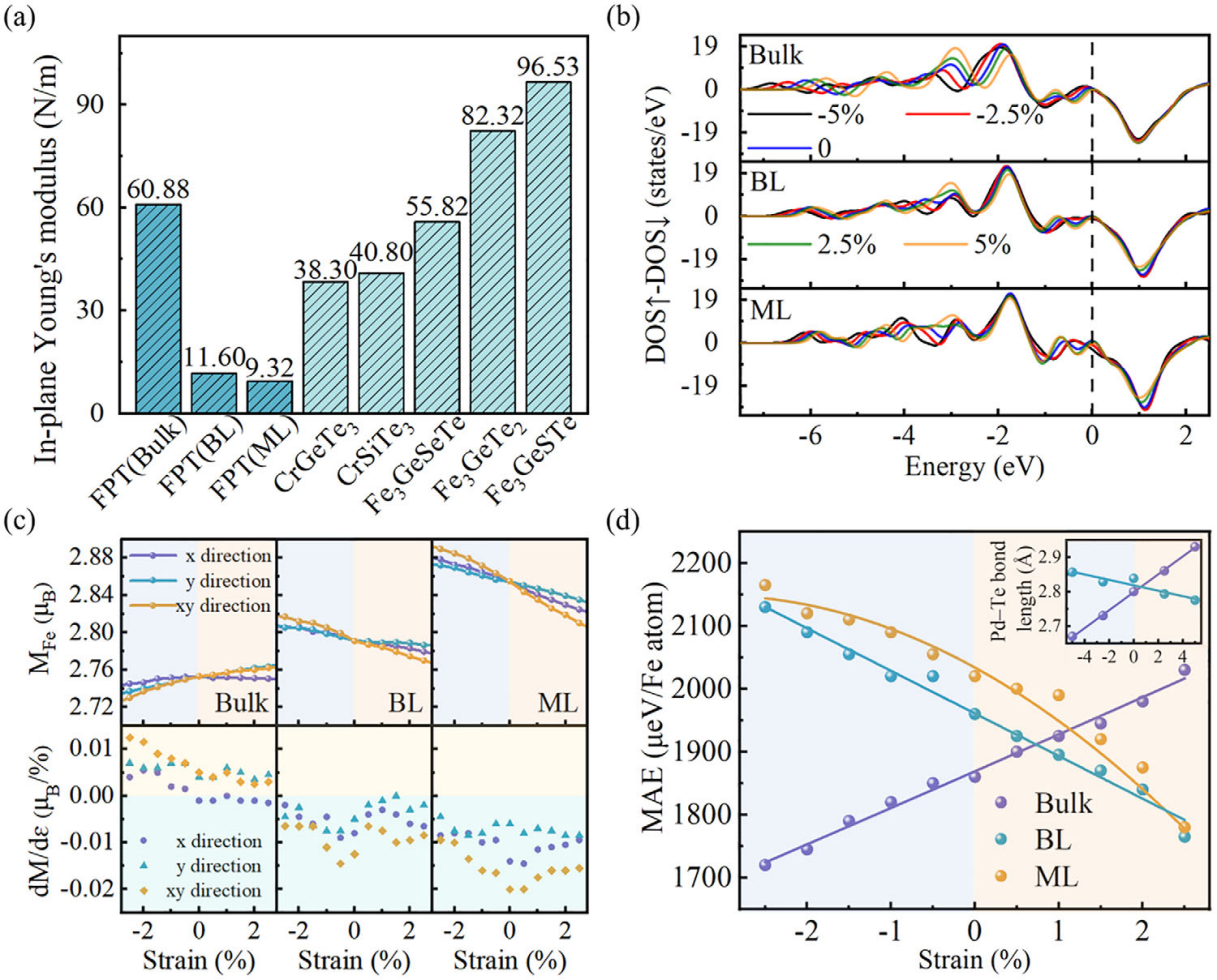
Dimensionality‐dependent magnetoelastic response of FPT. (a) In‐plane Young's modulus of FPT in bulk, BL, and ML form in comparison with several representative 2D magnets. (b) Strain‐modulated spin‐resolved DOS difference Δ*D*(*E*)  = *D*_↑_  − *D*_↓_ for bulk, BL, and ML FPT. (c) The top panels show the evolution of the M_Fe_ under in‐plane strain along the x, y, and xy directions for bulk, BL, and ML FPT. Bottom panels reveal strain derivatives dM/dε. (d) Variation in MAE as a function of in‐plane strain. Inset displays the evolution of the interlayer Pd─Te bond length as a function of strain.

The in‐plane stiffness of FPT was analyzed before the strain dependence of magnetism by obtaining the in‐plane 2D Young's moduli Y_2D_ from the anisotropic elastic tensor, as detailed in Section S3. The 2D Young's modulus Y_2D_ derived from the elastic constants (Methods) is summarized in Figure 4a. Overall, bulk FPT shows a moderate in‐plane stiffness of ∼60.9 N/m. After the breakage of the interlayer Pd─Te bridges, Y_2D_ drops to 11.6 N/m in the bilayer and 9.3 N/m in the monolayer, more than six times smaller than in the bulk. Bulk FPT therefore exhibits a moderate in‐plane stiffness, comparable to the lower end of Fe_3_GeTe_2_‐based 2D magnetic systems and somewhat larger than the typical monolayer values reported for CrGeTe_3_ and CrSiTe_3_ (∼38–41 N/m) [29]. Upon exfoliation, however, the bilayer and monolayer become markedly softer, rendering few‐layer FPT significantly more compliant than these representative vdW magnets and thus highly favorable for strain‐engineering applications.

The strain evolution of the spin‐polarized density of states, plotted as Δ*D*(*E*)  = *D*_↑_  − *D*_↓_, for bulk, bilayer, and monolayer FPT under biaxial strains from −5% to +5% is shown in Figure 4b. In bulk, both compressive and tensile strain visibly reshape ΔDOS, especially between −4 and −1 eV, indicating that the three‐dimensional Fe─Te─Pd network is highly sensitive to lattice distortion. The reduction in thickness strongly attenuates these changes. In the monolayer, the ΔDOS curves under different strains nearly overlap across the considered energy window, indicating that the near‐E_F_ spin polarization is comparatively insensitive to biaxial strain up to ±5%. This feature, however, should not be interpreted as implying a strain‐invariant local Fe moment, because the calculated dM/dε is still appreciable and even becomes larger in magnitude in the monolayer. In other words, dimensional reduction renders the spin‐polarized electronic structure near E_F_ comparatively robust against biaxial strain up to ±5%, while the local Fe moment and the MAE remain tunable under strain.

This contrast can be explained by how dimensionality reshapes the available hybridization channels. In monolayer FPT, the absence of Pd─Te interlayer bridges indicates magnetism governed by both the in‐plane Fe─Te─Fe paths and the crystal‐field splitting of the Fe‐d manifold. The application of moderate biaxial strain perturbs these in‐plane geometries only slightly, leading to nearly rigid spin splitting and only minor changes in ΔDOS near E_F_. By contrast, the sparse but significant Pd─Te bridges in bulk tie Fe layers into a three‐dimensional Fe─Te─Pd network. In this case, in‐plane strain modifies not only the Fe─Te─Fe paths but also the Pd─Te distances and angles through internal relaxations, strongly modulating orbital energies and hybridization strengths, confirming the strain dependence of the spin‐polarized DOS observed in Figure 4b.

The anomalous, dimension‐dependent magnetoelastic response of the Fe moments is presented in Figure 4c. In the bulk, the magnetic moment increases slightly under tensile strain (dM/dε>0), whereas a decrease is observed for both bilayer and monolayer (dM/dε<0), with the most negative slope recorded for the monolayer. The definition of dM/dε is given in Equation (S18), and the strain‐dependent values of M_Fe_ and dM/dε are provided in Tables S6 and S7. To facilitate comparison with the broader magnetoelastic literature, we further estimate an effective near‐equilibrium 2D piezomagnetic coefficient q_2D_ = dM/dσ_2D_ by converting the calculated strain derivative dM/dε using the effective in‐plane 2D modulus Y_2D_. Because the present calculations are strain‐controlled and carried out within a 2D elastic formalism, the resulting q_2D_ values should be regarded as estimated comparison metrics rather than directly measured bulk piezomagnetic coefficients. The estimated coefficients show a clear dimensional trend: the magnitude of the stress‐normalized magnetic response is much smaller in bulk FPT than in the bilayer and monolayer, indicating that the few‐layer limit combines much greater mechanical compliance with stronger magnetoelastic tunability. The corresponding q_2D_ values, together with the effective 3D converted values for auxiliary comparison, are summarized in Table S8. The Bader charge analysis in Figure S11 shows lengthening of the Pd─Te bonds and weakening of the three‐dimensional Fe─Te─Pd “vertical” network by tension in bulk, reducing charge loss on Fe and enhancing local spin polarization. After exfoliation, the same in‐plane strain either strengthens the remaining Pd─Te covalency in the bilayer or directly distorts the soft in‐plane Fe─Te─Pd polyhedra in the monolayer, promoting charge transfer away from Fe, thereby suppressing the magnetic moments of Fe ions.

In Figure 4d, an analogous sign reversal appears in the strain derivative of the MAE, with the angular dependence of MAE on strain for bulk, bilayer, and monolayer FPT detailed in Figures S12–S41. In bulk FPT, loosening the three‐dimensional Fe─Te–Pd network under tension narrows relevant orbital gaps and localizes Fe‐d states near E_F_, resulting in a modest but monotonic increase in MAE as a function of strain. By comparison, biaxial tension in the bilayer and monolayer reduces the near‐E_F_ weight of the d_z_^2^, d_xy_, and d_xz_ orbitals dominating the anisotropy, simultaneously lowering the Fe moments and leading to an almost linear decrease of MAE and the largest negative dMAE/dε in the monolayer (Figures S42–S44).

Taken together, these results indicate that the sparse Pd─Te covalent bridges not only control cleavage energetics but also open distinct magnetoelastic channels in the bulk and 2D limits. In bulk, strain primarily acts by loosening a Pd─Te–mediated three‐dimensional network, slightly localizing Fe‐d states, and increasing both M_Fe_ and MAE. After exfoliation, the same strain directly distorts ultracompliant Fe─Te─Pd polyhedra, enhancing covalency and strongly suppressing both the magnetic moment and the SOC‐driven anisotropy. Since this bulk‐to‐monolayer sign reversal in the strain derivatives of M_Fe_ and MAE is difficult to induce in canonical vdW magnets, these results indicate a distinct dimension‐dependent magnetoelastic response associated with the non‐vdW bonding topology of FPT.

## 3 Conclusions

The electronic structure and magnetism of FPT were thoroughly investigated by calibrated first‐principles calculations. The following key conclusions can be drawn:

1. FPT is shown to behave as a non‐vdW layered ferromagnet with sparse interlayer Pd─Te covalent bridges, and is associated with nontrivial thickness‐dependent evolution of the electronic structure and magnetism beyond a simple weak‐vdW picture. Despite the covalent nature of these bridges, their low density induces a cleavage energy (∼0.51 J·m^−2^) comparable to those of vdW crystals, facilitating the exfoliability of FPT.
2. The reduction from bulk to monolayer results in an unusual coexistence of slightly strengthened local magnetic moments and intralayer exchange terms together with markedly weakened long‐range magnetic order. Although J_1_ and |J_2_| increase moderately upon thinning, the suppression of T_C_ is governed primarily by the progressive weakening and eventual disappearance of interlayer ferromagnetic exchange J_3_, while the strengthening of antiferromagnetic J_2_ provides an additional destabilizing contribution. This behavior reflects a rebalancing of the exchange network rather than a simple weakening of interlayer coupling alone.
3. FPT exhibits a relatively large easy‐plane magnetocrystalline anisotropy of ∼1.86 meV per Fe in the bulk. This value increases by about 8.6% in the monolayer, due to intralayer Fe─Te─Pd coordination bringing d_z_^2^, d_xy_, and d_xz_ states closer to the Fermi level, thereby maintaining strong easy‐plane anisotropy despite the removal of interlayer exchange.
4. A dimensionality‐driven reversal is recorded in the magnetoelastic response, where tensile strain increases magnetization and anisotropy in the bulk but suppresses both in the monolayer, resulting in opposite signs of dM/dε and dMAE/dε. This reversal indicates a distinct dimension‐dependent magnetoelastic response associated with the non‐vdW bonding topology of FPT.

Overall, these results clarify how sparse interlayer covalent bridges govern the thickness‐dependent magnetic evolution of FPT, establish this material as a representative exfoliable non‐vdW layered magnet, and highlight the potential relevance of such bonding‐governed layered systems for ultrathin spintronic applications.

## 4 Methods

The first‐principles calculations were all performed within spin‐polarized density functional theory (DFT) as implemented in the Vienna Ab initio Simulation Package (VASP) [30, 31]. The interaction between valence electrons and ionic cores was described by the projector‐augmented‐wave (PAW) method [32], and exchange‐correlation effects were treated by the generalized‐gradient approximation (GGA) in the Perdew–Burke–Ernzerhof (PBE) parametrization [33, 34]. No Hubbard U correction was applied to Fe in the present work. This choice was made because FPT is not a prototypical localized Mott‐type Fe system; instead, its low‐energy electronic structure involves substantial Fe─Te─Pd hybridization. In addition, standard PBE already reproduces the experimentally reported lattice parameters, Fe local moment, and T_C_ reasonably well (Table S1). Preliminary tests further indicated that introducing a U term on Fe does not improve, and in fact tends to worsen, the description of the experimentally supported interlayer Pd─Te bridge geometry, which is central to the present study. We therefore adopted standard PBE to avoid over‐localizing the Fe‐3d states and to preserve a more realistic description of the Fe─Te─Pd bonding network. This treatment is also consistent with the original experimental/theoretical report on FePd_2_Te_2_ [14]. A plane‐wave kinetic‐energy cutoff of 500 eV was adopted for all calculations. For Brillouin‐zone integrations, Monkhorst‐Pack k‐point meshes of 5 × 5 × 3 were used for bulk FPT and 5 × 5 × 1 for the layered geometries, followed by verification to converge both the total energies and magnetic moments [35, 36, 37].

In the simulations, both bulk and few‐layer structures were fully relaxed until the residual Hellmann‐Feynman forces on each atom became smaller than 0.01 eV/Å and the total‐energy change between successive electronic steps was less than 10^−6^ eV. For selected calculations requiring higher numerical accuracy, such as MAE and cleavage energy, the electronic convergence threshold was tightened to 10^−7^ eV. For the layered geometries, a vacuum spacing of at least 20 Å was introduced along the out‐of‐plane direction to eliminate spurious interactions between periodic images. Also, all in‐plane lattice constants and internal coordinates were allowed to relax. Cleavage energies and bridge dissociation energies were calculated from total‐energy differences between appropriately cleaved or bond‐broken configurations and the corresponding intact reference structures, followed by normalization by either surface area or by the number of Pd─Te bridges.

All electronic‐structure analyses, including spin‐resolved densities of states, element‐ and orbital‐projected band structures, and charge‐density‐difference maps, were performed using post‐processing utilities for VASP [38, 39], and visualized with VESTA [40]. The interlayer Pd─Te bonding was further characterized by calculating electron localization function (ELF) maps on three‐dimensional grids [41]. The Curie temperature (T_C_) was evaluated using the Vampire atomistic spin package [42] together with Monte Carlo simulations to capture the finite‐temperature thermodynamic behavior of the system [43, 44]. Charge transfer among Fe, Pd, and Te atoms, as well as its evolution with dimensionality and strain, was quantified by Bader charge analysis [45, 46, 47].

The in‐plane elastic response and effective 2D Young's modulus were obtained by projecting the full elastic tensor under a plane‐stress condition, followed by the evaluation of the directional Young's modulus according to Equations (S15)–(S17).

## Author Contributions

**Jianwen Fang**: software. **Huaiyuan Zhao**: investigation, writing – original draft. **Mohan Luo**: investigation. **Yulin Wang**: formal analysis. **Yinuo Ye**: investigation. **Peng Cheng**: formal analysis. **Hongwen Zhang**: formal analysis. **Jianhui Yang**: supervision, conceptualization, writing – review and editing.

## Conflicts of Interest

The authors declare no conflicts of interest.

## Supporting information




**Supporting File**: advs75957‐sup‐0001‐SuppMat.docx.

## Data Availability

The data that support the findings of this study are available on request from the corresponding author. The data are not publicly available due to privacy or ethical restrictions.
