# Supplementary material for: Sparse Pd–Te Covalent Bridges Drive Anomalous Bulk‐to‐Monolayer Electronic and Magnetic Evolution in FePd2Te2
Source: Adv Sci (Weinh). 2026 Jun 3;13(48):e75957. doi: 10.1002/advs.75957 (PMC13336938; doi:10.1002/advs.75957)
Supplement: Supplementary file 1 [file ADVS-13-e75957-s001.docx]

Supporting Information

**Sparse Pd–Te Covalent Bridges Drive Anomalous Bulk-to-Monolayer Electronic and Magnetic** **Evolution in FePd_2_Te_2_**

*Huaiyuan Zhao ^1^, Jianwen Fang ^1^, Mohan Luo ^1^, Yinuo Ye ^1^, Yulin Wang ^1^, Jianhui Yang ^1, 2, *^, Hongwen Zhang ^3, *^ and* *Peng Cheng ^4^*

^1^ Quzhou University, Quzhou 324000, P. R. China

^2^ Key Laboratory of Advanced Fuel Cells and Electrolyzers Technology of Zhejiang Province, Ningbo Institute of Materials Technology and Engineering, Chinese Academy of Sciences, Ningbo, Zhejiang 315201, P. R. China

^3^ Key Lab of Materials Physics, Anhui Key Lab of Nanomaterials and Nanotechnology, Institute of Solid State Physics, HFIPS, Chinese Academy of Sciences, Hefei 230031, P. R. China

^4^ School of Physics, Beijing Key Laboratory of Opto-electronic Functional Materials & Micro-nano Devices, Renmin University of China, Beijing 100872, P. R. China

**Correspondence:** Jianhui Yang (jianhuiyoung@gmail.com), Hongwen Zhang(hwzhang@issp.ac.cn)

Table S1 丨 Experimental and calculated structural and magnetic parameters of bulk FePd_2_Te_2_. L_a_, L_b_, and L_c_ are the lattice constants; α, β, and γ are the lattice angles; M_Fe_ is the local magnetic moment on Fe; and T_C_ is the Curie temperature. The error rate is defined as (Cal. Val. −Exp. Val. )/Exp. Val. × 100%.

|  | Exp. Val. | Cal. Val. | Error Rate (％) |
| --- | --- | --- | --- |
| L_a_ | 7.50 Å | 7.53 Å | 0.4 |
| L_b_ | 3.95 Å | 3.98 Å | 0.8 |
| L_c_ | 7.74 Å | 7.83 Å | 1.2 |
| α | 90° | 90° | 0 |
| β | 118° | 118° | 0 |
| γ | 90° | 90° | 0 |
| M_Fe_ | 2.83 u_B_ | 2.75 u_B_ | -2.8 |
| T_C_ | 183 K | 173 K | -5.5 |

$\delta=\frac{x_{cal}-x_{exp}}{x_{exp}}$ Equation (S1)

Relative deviation between calculated and experimental values, where x_cal_ and x_exp_ denote the calculated and experimental quantities, respectively.

$\text{E}_{\text{dis}}^{\text{(}\text{per}\text{ }\text{bridge}\text{)}}\text{=}\frac{\text{E}_{\text{broken}}\text{-}\text{E}_{\text{intact}}}{\text{N}_{\text{bridge}}}$ Equation (S2)

Per-bridge dissociation energy, defined from the total-energy difference between the broken and intact configurations, where E_broken_ and E_intact_ are the corresponding total energies, and N_bridge_ is the number of bridges.

Figure S1 丨 Magnetic configurations of bulk FPT: FM, AFM1, AFM2, and AFM3 (left to right); arrows denote Fe spin directions.

Figure S2 丨 Magnetic configurations of bilayer (BL) FPT: FM, AFM1, AFM2, and AFM3 (left to right); arrows denote Fe spin directions.

Figure S3 丨 Magnetic configurations of monolayer (ML) FPT: FM, AFM1, and AFM2 (left to right); arrows denote Fe spin directions.

Table S2 丨 Optimized lattice parameters and absolute Fe magnetic moments M_Fe_ for bulk FePd_2_Te_2_ in the ferromagnetic (FM) and antiferromagnetic (AFM1–AFM3) configurations. L_a_, L_b_, and L_c_ are the lattice constants, and α, β, and γ are the lattice angles. The definitions of FM-AFM3 follow those in Figure S1.

|  | FM | AFM1 | AFM2 | AFM3 |
| --- | --- | --- | --- | --- |
| L_a_ | 7.92 | 7.88 | 7.94 | 7.97 |
| L_b_ | 7.96 | 7.94 | 8.03 | 8.11 |
| L_c_ | 13.17 | 13.27 | 13.13 | 13.03 |
| α | 90.00 | 90.00 | 90.12 | 90.00 |
| β | 87.35 | 87.32 | 87.41 | 88.08 |
| γ | 90.00 | 90.00 | 89.33 | 90.00 |
| \|M_Fe_\| | 2.75 | 2.77 | 2.73 | 2.62 |

Table S3 丨 Optimized lattice parameters and absolute Fe magnetic moments M_Fe_ for bilayer (BL) FePd_2_Te_2_ in the ferromagnetic (FM) and antiferromagnetic (AFM1–AFM3) configurations. L_a_, L_b_, and L_c_ are the lattice constants, and α, β, and γ are the lattice angles. The definitions of FM-AFM3 follow those in Figure S2.

|  | FM | AFM1 | AFM2 | AFM3 |
| --- | --- | --- | --- | --- |
| L_a_ | 7.88 | 7.86 | 7.85 | 7.87 |
| L_b_ | 7.89 | 7.97 | 7.99 | 8.04 |
| L_c_ | 30.71 | 30.71 | 30.71 | 30.71 |
| α | 90.00 | 90.00 | 90.00 | 90.00 |
| β | 90.00 | 90.00 | 90.00 | 90.00 |
| γ | 90.00 | 90.00 | 89.52 | 90.00 |
| \|M_Fe_\| | 2.79 | 2.83 | 2.77 | 2.71 |

Table S4 丨 Optimized lattice parameters and absolute Fe magnetic moments M_Fe_ for monolayer (ML) FePd_2_Te_2_ in the ferromagnetic (FM) and antiferromagnetic (AFM1, AFM2) configurations. L_a_, L_b_, and L_c_ are the lattice constants, and α, β, and γ are the lattice angles. The definitions of FM-AFM2 follow those in Figure S3.

|  | FM | AFM1 | AFM2 |
| --- | --- | --- | --- |
| L_a_ | 7.85 | 7.82 | 7.73 |
| L_b_ | 7.83 | 7.93 | 8.03 |
| L_c_ | 30.00 | 30.00 | 30.00 |
| α | 90.00 | 90.00 | 90.00 |
| β | 90.00 | 90.00 | 90.00 |
| γ | 90.00 | 89.61 | 90.00 |
| \|M_Fe_\| | 2.86 | 2.82 | 2.78 |

**Section S1 丨 Calculation of exchange coupling parameters J_1_, J_2_, and J_3_**

Heisenberg model was used to calculate the Curie temperature T_C_ of FPT. First, the exchange coupling parameters (J_1_, J_2_, and J_3_) were calculated, where J_1_, J_2_, and J_3_ represent the nearest, next-nearest, and next-next-nearest neighbor exchange coupling parameters, respectively, as is shown in Figure S4. The exchange interaction is given by:

$E_{ex}=-\sum_{i,j} J_{1}\boldsymbol{S}_{i}\cdot\boldsymbol{S}_{j}-\sum_{i,l} J_{2}\boldsymbol{S}_{i}\cdot\boldsymbol{S}_{l}-\sum_{i,k} J_{3}\boldsymbol{S}_{i}\cdot\boldsymbol{S}_{k}$ Equation (S3)

where S_i_ is the magnetic moment of Fe atoms.

Figure S4 丨 Exchange-coupling paths (J_1_-J_3_) used for Heisenberg mapping in FPT; arrows indicate Fe spin directions.

As shown in Figure S4, all Fe ions have parallel spins in the FM configuration; therefore, the total energy of the system can be written as Equation (S4). In the AFM1 configuration, all nearest- and next-nearest-neighbor Fe pairs are aligned ferromagnetically, whereas the next-next-nearest neighbors are antiferromagnetically aligned. In AFM2, the number of parallel/antiparallel Fe–Fe pairs per Fe site is 1/1 for nearest neighbors, 0/2 for next-nearest neighbors, and 2/2 for next-next-nearest neighbors, respectively. In AFM3, these numbers are 0/2, 2/0, and 0/4, respectively. On this basis, the Heisenberg Hamiltonian for the FM, AFM1, AFM2, and AFM3 configurations of FPT can be written as:

E_FM_ = E_0_ – (2J_1_ + 2J_2_ + 4J_3_)S^2^	Equation (S4)

E_AFM1_ = E_0_ – (2J_1_ + 2J_2_ – 4J_3_)S^2^	Equation (S5)

E_AFM2_ = E_0_ – (–2 J_2_ )S^2^	Equation (S6)

E_AFM3_ = E_0_ – (–2J_1_ + 2J_2_ – 4J_3_)S^2^	Equation (S7)

where S is the net magnetic moment of the Fe atom, and E_FM_, E_AFM1_, E_AFM2_, E_AFM3_ stand for the total energy of the FPT system in the FM, AFM1, AFM2, AFM3 configurations, respectively. E_0_ is the total energy of systems excluding exchange energy. From these four equations, J_1_, J_2_, and J_3_ can be calculated as follows:

J_1_ = (E_AFM3_ – E_AFM1_)/4S^2^	Equation (S8)

J_2_ = (E_AFM2_ – E_AFM3_ – E_FM_)/8S^2^	Equation (S9)

J_3_ = (E_AFM1_ – E_FM_)/8S^2^	Equation (S10)

Table S5 丨 Bare exchange constants and multiplicity-weighted exchange contributions for bulk, bilayer, and monolayer FPT.

| System | J_1_ (meV) | J_2_ (meV) | J_3_ (meV) | 2J_1_ (meV) | 2J_2_ (meV) | 4J_3_ (meV) | 2J_1_+2J_2_+4J_3_ (meV) | T_C_ (K) |
| --- | --- | --- | --- | --- | --- | --- | --- | --- |
| Bulk | 49.93 | -5.64 | 8.26 | 99.86 | -11.28 | 33.04 | 121.62 | 173 |
| BL | 51.46 | -8.33 | 5.44 | 102.92 | -16.66 | 21.76 | 108.02 | 83 |
| ML | 51.83 | -9.37 | 0 | 103.66 | -18.74 | 0.00 | 84.92 | 27 |

Figure S5 丨 Element-projected band structure of bulk FPT for the spin-up channel. Colors: Fe (purple), Pd (blue), Te (yellow); marker size encodes the projected weight (fractional contribution).

Figure S6 丨 Element-projected band structure of bulk FPT for the spin-down channel. Colors: Fe (purple), Pd (blue), Te (yellow); marker size encodes the projected weight (fractional contribution).

Figure S7 丨 Element-projected band structure of BL FPT for the spin-up channel. Colors: Fe (purple), Pd (blue), Te (yellow); marker size encodes the projected weight (fractional contribution).

Figure S8 丨 Element-projected band structure of BL FPT for the spin-down channel. Colors: Fe (purple), Pd (blue), Te (yellow); marker size encodes the projected weight (fractional contribution).

Figure S9 丨 Element-projected band structure of ML FPT for the spin-up channel. Colors: Fe (purple), Pd (blue), Te (yellow); marker size encodes the projected weight (fractional contribution).

Figure S10 丨 Element-projected band structure of ML FPT for the spin-down channel. Colors: Fe (purple), Pd (blue), Te (yellow); marker size encodes the projected weight (fractional contribution).

**Section S2 丨 PDOS-based orbital analysis of magnetic anisotropy**

To analyze how dimensionality reorganizes the spin–orbit–active Fe-d manifold near the Fermi energy and thereby affects the MAE, we quantify two complementary quantities from the projected density of states (PDOS): (i) an “orbital proximity” Δε(A→B) between selected occupied and unoccupied orbitals, which represents the effective level spacing in the denominator of Bruno’s expression, and (ii) a near-E_F_ spectral weight I_sum_(X) for each orbital X, which measures how many states are available for SOC-mediated virtual excitations.

For a given Fe-d orbital A, we define a PDOS-weighted energy centroid for the occupied states in a window [−0.4,0] eV relative to the Fermi energy,

${\overset{¯}{E}}_{occ}(A)=\frac{\int_{-0.4}^{0}ED_{A}^{tot}(E)dE}{\int_{-0.4}^{0}D_{A}^{tot}(E)dE}$ Equation (S11)

and, similarly, for an unoccupied orbital B in the window [0,0.4] eV,

${\overset{¯}{E}}_{unocc}(B)=\frac{\int_{0}^{0.4}ED_{B}^{tot}(E)dE}{\int_{0}^{0.4}D_{B}^{tot}(E)dE}$ Equation (S12)

Here D_X_^tot^(E)=D_X_↑(E)+∣D_X_↓(E)∣is the spin-summed PDOS of orbital X. The effective orbital energy separation entering Bruno’s denominator between an occupied orbital A and an unoccupied orbital B is then approximated as

$\Delta\varepsilon_{A\to B}={\overset{¯}{E}}_{unocc}(B)-{\overset{¯}{E}}_{occ}(A).$ Equation (S13)

Smaller values of Δε(A→B) indicate that the corresponding occupied and unoccupied states are closer in energy and hence contribute more strongly to the MAE through the (ε_u_−ε_o_)^−1^ factor in Bruno’s expression [1].

To characterize the amount of SOC-active spectral weight in the numerator of Bruno’s formula, we further define a near-E_F_ PDOS integral for each Fe-d orbital X,

$I_{sum}(X)=\int_{-0.4}^{0}D_{X}^{tot}(E)dE+\int_{0}^{0.4}D_{X}^{tot}(E)dE$ Equation (S14)

which counts the total spin-summed spectral weight of orbital X within ±0.4 eV of the E_F_. A larger I_sum_(X) implies that more states of orbital character X are available for SOC-mediated virtual excitations, and therefore that this orbital channel can make a stronger contribution to the MAE.

In the main text (Figure 3c–d), we use Δε(A→B) to construct the “orbital proximity” maps and I_sum_(X) to evaluate the near-E_F_ weight of the key d_z_^2^, d_xy_, and d_xz_ orbitals, thereby linking the dimensionality-induced reorganization of the Fe-d manifold to the observed enhancement of the easy-plane MAE in ultrathin FPT.

**Section S3 丨 Evaluation of in-plane elastic constants and 2D Young’s modulus**

The in-plane elastic response of FPT was evaluated using the full anisotropic elasticity formalism. Under the plane-stress condition, the effective in-plane stiffness tensor is constructed as [2]:

$\overset{\sim}{C}=C_{\alpha\alpha}-C_{\alpha k}C_{kk}^{-1}C_{k\alpha}$ Equation (S15)

This tensor is then inverted to obtain the in-plane compliance ${\overset{\sim}{S}}$.

The directional Young’s modulus in the xy-plane is then given by [3-4]:

$$\frac{1}{E\left( \theta\right)}={{\overset{\sim}{S}}_{11}cos}^{4} \theta+{{\overset{\sim}{S}}_{22}sin}^{4} \theta+\left( 2{\overset{}{{\overset{\sim}{S}}_{12}+S}}_{66} \right){cos}^{2} \theta{sin}^{2} \theta+2{\overset{\sim}{S}}_{16}{cos}^{3} \theta\sin\theta$$

$+2{\overset{\sim}{S}}_{26}{sin}^{3}\theta cos\theta$	Equation (S16)

The principal moduli are E_x_=1/${\overset{\sim}{S}}_{11}$ and E_y_=1/${\overset{\sim}{S}}_{22}$.

For comparison with 2D materials, the effective 2D modulus is defined as [5]:

$E_{2D}=E_{3D}t_{eff}$	Equation (S17)

where t_eff_ is the physical slab thickness (10.40 Å for bulk, 10.35 Å for the bilayer, and 3.98 Å for the monolayer).

Here dM/dε is obtained from a central finite difference.

$\frac{dM}{d\varepsilon}=\frac{M_{s+1}-M_{s-1}}{2\Delta\varepsilon}$ Equation (S18)

where M_s+1_ and M_s−1_ are the magnetic moments at strains ε_s_+Δε and ε_s_−Δε, respectively, and Δε is the strain increment.

Table S6 丨 Spin magnetic moments M_Fe_ (in μ_B_) of Fe in bulk, bilayer (BL), and monolayer (ML) FePd_2_Te_2_ under biaxial in-plane strain ε. Columns labeled x, y, and xy denote magnetization along the [100], [010], and [110] directions, respectively; positive (negative) strain corresponds to tension (compression).

| M_Fe_ (μ_B_) | | | | | | | | | | | |
| --- | --- | --- | --- | --- | --- | --- | --- | --- | --- | --- | --- |
| Bulk | | | | BL | | | | ML | | | |
| strain | x | y | xy | strain | x | y | xy | strain | x | y | xy |
| -5 | 2.7265 | 2.7180 | 2.6845 | -5 | 2.8130 | 2.8090 | 2.8180 | -5 | 2.9045 | 2.8735 | 2.9190 |
| -4.5 | 2.7320 | 2.7215 | 2.6985 | -4.5 | 2.8140 | 2.8090 | 2.8170 | -4.5 | 2.8980 | 2.8740 | 2.9130 |
| -4 | 2.7345 | 2.7255 | 2.7085 | -4 | 2.8090 | 2.8110 | 2.8195 | -4 | 2.8910 | 2.8745 | 2.9075 |
| -3.5 | 2.7395 | 2.7290 | 2.7170 | -3.5 | 2.8075 | 2.8090 | 2.8210 | -3.5 | 2.8855 | 2.8735 | 2.9005 |
| -3 | 2.7425 | 2.7330 | 2.7245 | -3 | 2.8080 | 2.8095 | 2.8185 | -3 | 2.8810 | 2.8740 | 2.8945 |
| -2.5 | 2.7450 | 2.7370 | 2.7300 | -2.5 | 2.8055 | 2.8055 | 2.8170 | -2.5 | 2.8785 | 2.8720 | 2.8895 |
| -2 | 2.7465 | 2.7400 | 2.7370 | -2 | 2.8060 | 2.8050 | 2.8120 | -2 | 2.8725 | 2.8695 | 2.8850 |
| -1.5 | 2.7505 | 2.7430 | 2.7415 | -1.5 | 2.8010 | 2.8030 | 2.8105 | -1.5 | 2.8705 | 2.8645 | 2.8795 |
| -1 | 2.7515 | 2.7460 | 2.7460 | -1 | 2.8000 | 2.7985 | 2.8055 | -1 | 2.8645 | 2.8610 | 2.8715 |
| -0.5 | 2.7525 | 2.7500 | 2.7495 | -0.5 | 2.7965 | 2.7955 | 2.7995 | -0.5 | 2.8605 | 2.8565 | 2.8635 |
| 0 | 2.7530 | 2.7530 | 2.7530 | 0 | 2.7910 | 2.7910 | 2.7910 | 0 | 2.8550 | 2.8550 | 2.8550 |
| 0.5 | 2.7515 | 2.7540 | 2.7545 | 0.5 | 2.7885 | 2.7905 | 2.7870 | 0.5 | 2.8465 | 2.8505 | 2.8435 |
| 1 | 2.7520 | 2.7570 | 2.7570 | 1 | 2.7870 | 2.7890 | 2.7845 | 1 | 2.8405 | 2.8470 | 2.8350 |
| 1.5 | 2.7515 | 2.7600 | 2.7595 | 1.5 | 2.7855 | 2.7895 | 2.7795 | 1.5 | 2.8350 | 2.8435 | 2.8260 |
| 2 | 2.7510 | 2.7620 | 2.7600 | 2 | 2.7830 | 2.7890 | 2.7745 | 2 | 2.8295 | 2.8395 | 2.8190 |
| 2.5 | 2.7505 | 2.7635 | 2.7620 | 2.5 | 2.7795 | 2.7865 | 2.7700 | 2.5 | 2.8245 | 2.8350 | 2.8100 |
| 3 | 2.7495 | 2.7665 | 2.7630 | 3 | 2.7765 | 2.7870 | 2.7660 | 3 | 2.8200 | 2.8310 | 2.8035 |
| 3.5 | 2.7500 | 2.7685 | 2.7640 | 3.5 | 2.7705 | 2.7870 | 2.7595 | 3.5 | 2.8170 | 2.8275 | 2.7970 |
| 4 | 2.7490 | 2.7715 | 2.7650 | 4 | 2.7685 | 2.7855 | 2.7545 | 4 | 2.8130 | 2.8250 | 2.7890 |
| 4.5 | 2.7500 | 2.7745 | 2.7660 | 4.5 | 2.7670 | 2.7865 | 2.7495 | 4.5 | 2.8095 | 2.8205 | 2.7815 |
| 5 | 2.7485 | 2.7765 | 2.7660 | 5 | 2.7635 | 2.7855 | 2.7440 | 5 | 2.8080 | 2.8170 | 2.7710 |

Table S7 丨 Numerical strain derivatives of the Fe magnetic moments, dM/dε (in μ_B_/%), for bulk, bilayer (BL), and monolayer (ML) FePd_2_Te_2_ under biaxial in-plane strain, obtained from the M_Fe_ data in Table S6.

| dM/dε (μ_B_/%) | | | | | | | | | | | |
| --- | --- | --- | --- | --- | --- | --- | --- | --- | --- | --- | --- |
| Bulk | | | | BL | | | | ML | | | |
| strain | x | y | xy | strain | x | y | xy | strain | x | y | xy |
| -5 | 0.0110 | 0.0070 | 0.0280 | -5 | 0.0020 | 0.0000 | -0.0020 | -5 | -0.0130 | 0.0010 | -0.0120 |
| -4.5 | 0.0080 | 0.0075 | 0.0240 | -4.5 | -0.0040 | 0.0020 | 0.0015 | -4.5 | -0.0135 | 0.0010 | -0.0115 |
| -4 | 0.0075 | 0.0075 | 0.0185 | -4 | -0.0065 | 0.0000 | 0.0040 | -4 | -0.0125 | -0.0005 | -0.0125 |
| -3.5 | 0.0080 | 0.0075 | 0.0160 | -3.5 | -0.0010 | -0.0015 | -0.0010 | -3.5 | -0.0100 | -0.0005 | -0.0130 |
| -3 | 0.0055 | 0.0080 | 0.0130 | -3 | -0.0020 | -0.0035 | -0.0040 | -3 | -0.0070 | -0.0015 | -0.0110 |
| -2.5 | 0.0040 | 0.0070 | 0.0125 | -2.5 | -0.0020 | -0.0045 | -0.0065 | -2.5 | -0.0085 | -0.0045 | -0.0095 |
| -2 | 0.0055 | 0.0060 | 0.0115 | -2 | -0.0045 | -0.0025 | -0.0065 | -2 | -0.0080 | -0.0075 | -0.0100 |
| -1.5 | 0.0050 | 0.0060 | 0.0090 | -1.5 | -0.0060 | -0.0065 | -0.0065 | -1.5 | -0.0080 | -0.0085 | -0.0135 |
| -1 | 0.0020 | 0.0070 | 0.0080 | -1 | -0.0045 | -0.0075 | -0.0110 | -1 | -0.0100 | -0.0080 | -0.0160 |
| -0.5 | 0.0015 | 0.0070 | 0.0070 | -0.5 | -0.0090 | -0.0075 | -0.0145 | -0.5 | -0.0095 | -0.0060 | -0.0165 |
| 0 | -0.0010 | 0.0040 | 0.0050 | 0 | -0.0080 | -0.0050 | -0.0125 | 0 | -0.0140 | -0.0060 | -0.0200 |
| 0.5 | -0.0010 | 0.0040 | 0.0040 | 0.5 | -0.0040 | -0.0020 | -0.0065 | 0.5 | -0.0145 | -0.0080 | -0.0200 |
| 1 | 0.0000 | 0.0060 | 0.0050 | 1 | -0.0030 | -0.0010 | -0.0075 | 1 | -0.0115 | -0.0070 | -0.0175 |
| 1.5 | -0.0010 | 0.0050 | 0.0030 | 1.5 | -0.0040 | 0.0000 | -0.0100 | 1.5 | -0.0110 | -0.0075 | -0.0160 |
| 2 | -0.0010 | 0.0035 | 0.0025 | 2 | -0.0060 | -0.0030 | -0.0095 | 2 | -0.0105 | -0.0085 | -0.0160 |
| 2.5 | -0.0015 | 0.0045 | 0.0030 | 2.5 | -0.0065 | -0.0020 | -0.0085 | 2.5 | -0.0095 | -0.0085 | -0.0155 |
| 3 | -0.0005 | 0.0050 | 0.0020 | 3 | -0.0090 | 0.0005 | -0.0105 | 3 | -0.0075 | -0.0075 | -0.0130 |
| 3.5 | -0.0005 | 0.0050 | 0.0020 | 3.5 | -0.0080 | -0.0015 | -0.0115 | 3.5 | -0.0070 | -0.0060 | -0.0145 |
| 4 | 0.0000 | 0.0060 | 0.0020 | 4 | -0.0035 | -0.0005 | -0.0100 | 4 | -0.0075 | -0.0070 | -0.0155 |
| 4.5 | -0.0005 | 0.0050 | 0.0010 | 4.5 | -0.0050 | 0.0000 | -0.0105 | 4.5 | -0.0050 | -0.0080 | -0.0180 |
| 5 | -0.0030 | 0.0040 | 0.0000 | 5 | -0.0070 | -0.0020 | -0.0110 | 5 | -0.0030 | -0.0070 | -0.0210 |

The effective near-equilibrium 2D piezomagnetic coefficient was evaluated as

$q_{eff}^{2D}\approx\frac{(dM/d\varepsilon)_{\varepsilon\approx0}}{Y^{2D}}$ Equation (S19)

where (dM/dε)_ε≈0_ was taken from **Table S7** at zero strain and converted from μ_B_/% to μ_B_ by multiplying by 100. Here Y_2D_ denotes the effective in-plane 2D modulus used in the main text. For auxiliary comparison with conventional stress-based quantities, the corresponding effective 3D coefficients were further obtained as

$q^{3D,eff}=q_{eff}^{2D}\times t_{eff}$	Equation (S20)

where t_eff_ is the effective slab thickness defined in **Equation (S17)**. These quantities are introduced as effective comparison metrics within the present strain-controlled 2D framework.

Table S8 丨 Effective near-equilibrium 2D piezomagnetic coefficients of bulk, BL, and ML FPT derived from the strain derivative of the local Fe moment and the effective in-plane 2D modulus. The corresponding effective 3D converted values, obtained using the slab thickness t_eff_, are also listed for auxiliary comparison.

| System | Y_2D_ (N/m) | t_eff_ (Å) | q_x,2D_  [μ_B_/(N·m^−1^)] | q_y,2D_  [μ_B_/(N·m^−1^)] | q_xy,2D_  [μ_B_/(N·m^−1^)] | q_x,3D_  [μ_B_/MPa] | q_y,3D_  [μ_B_/MPa] | q_xy,3D_  [μ_B_/MPa] |
| --- | --- | --- | --- | --- | --- | --- | --- | --- |
| Bulk | 60.9 | 10.4 | -0.00164 | 0.00657 | 0.00821 | -1.71×10^-6^ | 6.83×10^-6^ | 8.54×10^-6^ |
| BL | 11.6 | 10.35 | -0.06897 | -0.0431 | -0.10776 | -7.14×10^-5^ | -4.46×10^-5^ | -1.12×10^-4^ |
| ML | 9.3 | 3.98 | -0.15054 | -0.06452 | -0.21505 | -5.99×10^-5^ | -2.57×10^-5^ | -8.56×10^-5^ |

Figure S11 丨 Fe charge transfer Δq (e) versus in-plane strain ε for bulk (red squares), bilayer (blue circles), and monolayer (green triangles) FPT; solid lines represent linear fits.

Figure S12 丨 Angular dependence of MAE in bulk FPT under -2.5% strain with magnetization rotation in the xz plane.

Figure S13 丨 Angular dependence of MAE in bulk FPT under -2% strain with magnetization rotation in the xz plane.

Figure S14 丨 Angular dependence of MAE in bulk FPT under -1.5% strain with magnetization rotation in the xz plane.

Figure S15 丨 Angular dependence of MAE in bulk FPT under -1% strain with magnetization rotation in the xz plane.

Figure S16 丨 Angular dependence of MAE in bulk FPT under -0.5% strain with magnetization rotation in the xz plane.

Figure S17 丨 Angular dependence of MAE in bulk FPT under 0.5% strain with magnetization rotation in the xz plane.

Figure S18 丨 Angular dependence of MAE in bulk FPT under 1% strain with magnetization rotation in the xz plane.

Figure S19 丨 Angular dependence of MAE in bulk FPT under 1.5% strain with magnetization rotation in the xz plane.

Figure S20 丨 Angular dependence of MAE in bulk FPT under 2% strain with magnetization rotation in the xz plane.

Figure S21 丨 Angular dependence of MAE in bulk FPT under 2.5% strain with magnetization rotation in the xz plane.

Figure S22 丨 Angular dependence of MAE in BL FPT under -2.5% strain with magnetization rotation in the xz plane.

Figure S23 丨 Angular dependence of MAE in BL FPT under -2% strain with magnetization rotation in the xz plane.

Figure S24 丨 Angular dependence of MAE in BL FPT under -1.5% strain with magnetization rotation in the xz plane.

Figure S25 丨 Angular dependence of MAE in BL FPT under -1% strain with magnetization rotation in the xz plane.

Figure S26 丨 Angular dependence of MAE in BL FPT under -0.5% strain with magnetization rotation in the xz plane.

Figure S27 丨 Angular dependence of MAE in BL FPT under 0.5% strain with magnetization rotation in the xz plane.

Figure S28 丨 Angular dependence of MAE in BL FPT under 1% strain with magnetization rotation in the xz plane.

Figure S29 丨 Angular dependence of MAE in BL FPT under 1.5% strain with magnetization rotation in the xz plane.

Figure S30 丨 Angular dependence of MAE in BL FPT under 2% strain with magnetization rotation in the xz plane.

Figure S31 丨 Angular dependence of MAE in BL FPT under 2.5% strain with magnetization rotation in the xz plane.

Figure S32 丨 Angular dependence of MAE in ML FPT under -2.5% strain with magnetization rotation in the xz plane.

Figure S33 丨 Angular dependence of MAE in ML FPT under -2% strain with magnetization rotation in the xz plane.

Figure S34 丨 Angular dependence of MAE in ML FPT under -1.5% strain with magnetization rotation in the xz plane.

Figure S35 丨 Angular dependence of MAE in ML FPT under -1% strain with magnetization rotation in the xz plane.

Figure S36 丨 Angular dependence of MAE in ML FPT under -0.5% strain with magnetization rotation in the xz plane.

Figure S37 丨 Angular dependence of MAE in ML FPT under 0.5% strain with magnetization rotation in the xz plane.

Figure S38 丨 Angular dependence of MAE in ML FPT under 1% strain with magnetization rotation in the xz plane.

Figure S39 丨 Angular dependence of MAE in ML FPT under 1.5% strain with magnetization rotation in the xz plane.

Figure S40 丨 Angular dependence of MAE in ML FPT under 2% strain with magnetization rotation in the xz plane.

Figure S41 丨 Angular dependence of MAE in ML FPT under 2.5% strain with magnetization rotation in the xz plane.

Figure S42 丨 Fe d_z_^2^-projected DOS of FPT under in-plane strain: bulk (top), bilayer (middle), monolayer (bottom).

Figure S43 丨 Fe d_xy_-projected DOS of FPT under in-plane strain: bulk (top), bilayer (middle), monolayer (bottom).

Figure S44 丨 Fe d_xz_-projected DOS of FPT under in-plane strain: bulk (top), bilayer (middle), monolayer (bottom).

1.	P. Bruno, “Tight-Binding Approach to the Orbital Magnetic-Moment and Magnetocrystalline Anisotropy of Transition-Metal Monolayers,” *Physical Review B* 39, no. 1 (1989): 865-868.

2.	R. Hill, “The Elastic Behaviour of a Crystalline Aggregate,” *Proceedings of the Physical Society of London Section A* 65, no. 389 (1952): 349-355.

3.	X. F. Liu, Z. F. Zhang, Z. Ding, et al., “Highly Anisotropic Electronic and Mechanical Properties of Monolayer and Bilayer As_2_S_3_,” *Applied Surface Science* 542, no. 148665 (2021): 148665.

4.	S. Yalameha, Z. Nourbakhsh, D. Vashaee, “ELATooLs: A tool for Analyzing Anisotropic Elastic Properties of the 2D and 3D Materials,” *Computer Physics Communications* 288, no. 108728 (2023): 108195.

5.	C. Rong, T. Su, Z. K. Li, et al., “Elastic Properties and Tensile Strength of 2D Ti_3_C_2_T_x_ MXene Monolayers,” *Nature Communications* 15, no. 1 (2024): 1566.
